# The unexpected and the non-fitting – considering the edges of simulation as social practice

**DOI:** 10.1186/s41077-020-0120-y

**Published:** 2020-02-05

**Authors:** Peter Dieckmann

**Affiliations:** 1 Copenhagen Academy for Medical Education and Simulation (CAMES), Center for Human Resources and Education, Capital Region of Denmark, Herlev Hospital, 25.floor, Borgmester Ib Juuls Vej 1, 2730 Herlev, Denmark; 2 Department of Quality and Health Technology, Faculty of Health Sciences, University of Stavanger, Stavanger, Norway; 3 Department of Clinical Medicine, Copenhagen University, Copenhagen, Denmark

The headache is surprisingly strong and of a quality that is so different from all I knew before. The start was sudden and then it did not go away. “Tumor?”, I think; “Tension in the muscle”, my wife says. “Bleeding, Aneurism?”, I think. “Look at these nice videos showing relaxation exercises”, my wife says. So: what to do? Emergency room or finding the yoga mat?

This example sheds light on what I call the edges of simulation as social practice. In a social practice, people interact with each other and with their environment according to official and unofficial rules [1]. I call the interactions that are done by many and/or often the center of the social practice and interactions that occur seldomly or involve only few members of the social practice as the edges. These edges warrant attention even though they concern “only” few. Stakeholders in the in center of simulation as a social practice are, for example, course participants, commissioners (the people who “buy” courses or other activities), or colleagues of your course participants in clinical practice.

In this editorial, I foreground those aspects and stakeholders of simulation as social practice that are at the edges. This might shed new light on what “we” as simulation community do and also what we do not yet do. What did we consider in detail and what did we forget so far? This understanding will help us to do more complete evaluations of simulation practice that also look beyond what we expect to find. History is full of examples for such unexpected “side” effects of social practices [2]. As a researcher, I am simply curious to find out more about what we do.

So, I ask: What are the effects of the use of simulation beyond what we expect and hope to achieve? Who is affected by these simulations, and in what way?

The work around simulated and standardized patients (and other roles) is a good example of an area of simulation as social practice that asked similar questions and where a lot of progress was made to understand its impact on the different people involved [3–6]. For example: Confederates portraying a depressive patient, receiving bad news, acting as a relative in grief might be impacted by their role, and might need to be released from their role explicitly and debriefed separately [7–10].

I will provide illustrative examples of other stakeholders and practices in simulation as a social practice who might benefit from a similar focus. I hope to inspire more systematic research around those stakeholders and practices.

## Considering people on the edges of simulation as social practice

Let me start with the vignette in the beginning of this text. Working as a psychologist with healthcare simulation for almost 20 years, I have seen countless scenarios and debriefings, most bringing participants to the edge of their abilities. Consequently, I acquired an understanding of healthcare that is biased. I lack the palpable experiences of all the positive cases that go just fine, as the vast majority of the simulation cases I saw, did not go just fine. Therefore, subjectively, healthcare is much more risky to me than what statistics would suggest. I can find several known biases that could explain my perception [11], but those explanations do not take away the tension. I experience my biased view on healthcare as a burden—especially in the light of any symptoms of my own or those for whom I care (see my example in the start). I see an unexpected effect of simulation here: those working within healthcare, without a healthcare background, might be more anxious when interacting with the healthcare system than we should be, and I wonder who else is affected by simulation in unexpected ways.

Simulation educators described how their professional self-perception and status with colleagues changed, after leaving clinical work for a simulation career, an effect to take into account, when planning a career in simulation. You might gain in status as educator, and you might lose in status as clinician [12].

Many people are stressed when going into simulation [13]. For some, this is much more intense than expected as the simulator might trigger their “clown-phobia” [14, 15].

Most scenarios that I saw involved patients commonly seen in clinical practice (even though they might present seldom diseases). I saw much less scenarios that involved “less common” patients. Consider, for example, transgender patients. Involving them in scenarios, simulation could contribute to raise awareness of special needs of this patient group [16–18]. Such scenarios can create relevant learning on an organizational plane: how is the information about special needs of a patient passed on through the system? They also pose interesting challenges in terms of creating believable scenarios? What other patient groups would be interesting to involve in simulation to help prepare learners for diversity?

## Considering unexpected effects of simulation as social practice

In the following, I will describe some examples, where simulation generated effects that I found surprising, and that were—that is my assumption—not intended by those running these simulations.

Healthcare professionals, who were certified in advanced life support, described that they found it hard to work with people who were not certified. Course completion created unintended barriers for collaboration [19].

In the excitement of the benefits of in situ simulation, it took a while to recognize related safety challenges and ways to mitigate related risks [20–22].

Two course participants were traveling in the elevator to attend a simulation course. Unknown to them, a member of the simulation center rode along and told their tale. When expressing nervousness about the simulation course, one participants got the tip from the other: “Just say ‘closed loop communication’,” and they will be happy. It could be a side effect of insisting on using pre-defined communication techniques and terms that participants use the words, but might not connect to the meaning [23]. Educators and participants might share an illusion of competence, if participants use the right words. Simulation-based learning might stay “simulation-based” and might be challenged to connect to clinical practice [24, 25]. This surprise points to the need to balance the need for conceptual clarity with the wish to create an enjoyable experience for participants. Conceptual clarity would require potentially tiring discussions around details; enjoyable experiences might be fostered by accepting language that is in the right direction, but far from precise. It also points to the need to link simulation-based learning with workplace-based learning.

Consider a team calling for help during a simulation scenario (a very common learning goal in scenarios). What is likely to happen? They will get to know that help is on the way—but help will likely not arrive during the scenario. In many cases, there is simply no help to be sent in all participants that are involved in the scenario, and neither the simulation operator nor the educator can leave their post. Calling for help does not do any good, really, in such a case. It would be a big problem, if participants learn that calling for help does not really help in simulation practice and reconstruct it in clinical practice.

Or consider confederates within scenarios [8, 26]—often a nurse, actually part of the simulation team, who works with a group of physicians. I frequently saw this nurse being instructed to “just do as you are told,” “wait for instructions,” or “do not be too pro-active.” This makes great debriefing points: how do you activate a team member. I wonder, however, what kind of messages participants get in such scenarios between the lines: “do not count on your team members, they will not be very pro-active” [27]. Participants might learn how to “activate” such a team member, but do they learn how to work with one, who is really good at her or his job, who brings in ideas, who might even be more skilled in the particular situation than yourself?

About 7 years ago, I discovered that whenever we had surgeons in faculty development courses, who were friendly, kind, and constructive people, that I was surprised. I had no bad previous experiences with any surgeon; I had no experience with a surgeon outside of these courses at all. So, how could it be that I had an emotional “connection” to them? Upon reflection, I came up with this explanation: by that time, I had worked for about 12 years mainly with anesthesiologists and heard quite a share of stories and jokes about surgeons. Somehow, I must have ingrained a stereotype that I find a challenge for patient safety. If I—as an outsider—get ingrained with such a stereotype, what happens to the insiders? Since then, I worked with my stereotypes and I think my “assessments” of participants of the surgical profession are more fine-grained now.

## What that all means for simulation practice

All of these examples concern different “edges” of simulation as social practice. They hold big potential to bring in new perspectives on learning on the individual, team based, and organizational level. It might be that by focusing these edges, we find undiscovered keys to progress in simulation practice and with the wanted outcomes of improving safety and quality of care for patients and their relatives, as well as for improving the work conditions of health professionals. It might also be that we find new insights about the uses of simulation that do not fulfill what they seem to promise.

Simulations should be evaluated based on the effects that they should enable, for example, individual learning, changes in organizational processes, or effects on patients of these changes. Evaluations should also address unexpected effects and easy forgotten stakeholders [28, 29]. To really understand how simulation as a social practice works, evaluations would need to look beyond the immediate question of whether “it worked” [30]. Are simulation effects good effects or negative effects—and for whom? Are we overlooking effects or people, who might be affected by them? Do they occur now or maybe just over time? What are messages that are sent between the lines and that might not advance the case of simulation, of safety, of quality, of work satisfaction, etc.? Or are there other messages we are too busy, too enthusiastic, and too lazy to see, to hear, and to take seriously? We should become aware of possible biases that we reinforce, when we do need analyses, design courses and scenarios, debrief participants, evaluate activities, and report back to commissioners. The more mature simulation becomes as social practice, the more important it seems to investigate it from different angles. Becoming aware of the jokes told, the stories around virtual (and actual) fireplaces, in elevators, to loved ones at home, to strangers in bars—time to consider warnings given, feelings shared, and hopes uttered. What do they tell us about simulation as a social practice and how can we use them to limit the risk of harming anyone and to create the best value for patients, their relatives, and those who care for them?

## Data Availability

This editorial does not present any data and material as such. Therefore, no more pieces are available.
